# Regulation of sharp wave-ripples by cholecystokinin-expressing interneurons and parvalbumin-expressing basket cells in the hippocampal CA3 region

**DOI:** 10.3389/fncom.2025.1591003

**Published:** 2025-05-26

**Authors:** Yuchen Yang, Xiaojuan Sun

**Affiliations:** ^1^School of Mathematical Sciences, Beijing University of Posts and Telecommunications, Beijing, China; ^2^School of Physical Science and Technology, Beijing University of Posts and Telecommunications, Beijing, China; ^3^Key Laboratory of Mathematics and Information Networks (Beijing University of Posts and Telecommunications), Ministry of Education, Beijing, China

**Keywords:** sharp wave-ripples, interneurons, mutual inhibition, CA3, hippocampus

## Abstract

To explore the individual and interactive effects of the interneurons cholecystokinin-expressing interneurons (CCKs) and parvalbumin-expressing basket cells (BCs) on sharp wave-ripples (SWR) and the underlying mechanisms, we constructed a mathematical model of the hippocampal CA3 network. By modulating the activity of CCKs and BCs, it was verified that CCKs inhibit the generation of SWR, while the activity of BCs affects the occurrence of SWR. Additionally, it was postulated that CCKs exert an influence on SWR through a direct mechanism, wherein CCKs directly modulate pyramidal cells (PCs). It was also discovered that BCs control SWR mainly through mutual inhibition among BCs. Furthermore, by adjusting the strength of the interaction between BCs and CCKs at various levels, it was identified that the interaction between these two types of interneurons has a relatively symmetrical effect on the regulation of SWR, functioning through a mutual inhibition mechanism. Our findings not only offer a deeper understanding of how CCKs and BCs independently regulate the generation of SWR but also provide novel insights into how changes in the strength of their interaction affect network oscillations. The results emphasize the crucial role of inhibitory interneurons in maintaining normal hippocampal oscillations, which are essential for proper brain function, particularly in the domains of memory consolidation and cognitive processes.

## 1 Introduction

The brain generates a variety of oscillatory rhythms that are closely linked to distinct cognitive functions (Doelling and Assaneo, 2021). Among these, sharp-wave ripples (SWRs) constitute one of the most synchronous patterns (Csicsvari et al., 1999). In rodents, SWRs occur within a frequency range of 120-250 Hz and are prominently observed during quiet wakefulness and slow-wave sleep. These events are associated with the replay of neural activity sequences and are regarded as playing a key role in memory consolidation and learning (Ólafsdóttir et al., 2018; Fernández-Ruiz et al., 2019). Disruptions in SWR activity are frequently associated with substantial memory impairments, which may contribute to the onset of various memory-related disorders (Oliva et al., 2020; Ego-Stengel and Wilson, 2010). It is increasingly evident that the normal generation of SWR oscillations depends on the dynamic interactions between excitatory and inhibitory neurons (Klausberger and Somogyi, 2008; Fishell and Kepecs, 2020). Thus, understanding the contributions of specific types of neurons to particular oscillatory patterns is crucial for uncovering the mechanisms underlying brain function (Kepecs and Fishell, 2014; McBain and Fisahn, 2001).

Experimental studies have demonstrated that CA3 pyramidal cells (PCs) exhibit synchronized burst activity during sharp waves, which subsequently propagate to the CA1 region and elicit high-frequency ripple oscillations (Buzsáki, 1986). Sharp wave-ripples represent a composite waveform resulting from the superposition of sharp waves and ripples (Buzsáki, 2015). Although ripples are most pronounced in the CA1 region, the CA3 region is essential for generating sharp waves, which act as the primary driver of SWR oscillations (Levy, 1996; Káli and Dayan, 2000; Davoudi and Foster, 2019). Therefore, we hypothesize that the CA3 region plays a critical role in SWR generation. Furthermore, it harbors excitatory recurrent networks that facilitate the storage and retrieval of brain activity patterns (McNaughton and Morris, 1987). In 2018, Hunt et al. discovered functional heterogeneity within the PC population of the hippocampal CA3, identifying a novel type of athorny pyramidal cells (athorny PCs), which differs both functionally and morphologically from the traditional thorny pyramidal cells (thorny PCs). A crucial distinction between these two neuron types lies in their firing patterns: thorny PCs exhibit a regular-spike firing pattern, while athorny PCs display burst firing (Hunt et al., 2018). Recent studies have demonstrated that athorny PCs can promote the occurrence of SWRs. The firing of these neurons contributes to the synchronized firing of thorny PCs, further supporting their involvement in SWR generation (Yang et al., 2024). Thus, we propose that both thorny and athorny PCs are indispensable excitatory components for the generation of SWRs.

In addition to excitatory neurons, the involvement of interneurons is critical for the proper generation of SWRs. Studies have indicated that the activity of parvalbumin-expressing basket cells (BCs) and cholecystokinin-expressing interneurons (CCKs) is closely associated with the duration and behavior of SWRs during hippocampal learning. Specifically, CCKs are known to shape the inhibitory tone prior to SWR events, playing a key role in modulating the duration of SWRs, while BCs provide inhibitory feedback that governs the normal oscillatory activity of the network (Vancura et al., 2023; Kohus et al., 2016; Klausberger et al., 2005). Therefore, we hypothesize that BCs and CCKs play an important role in the regulation of SWRs and that their interactions are crucial for controlling SWR dynamics. However, the specific mechanisms by which different types of interneurons modulate SWRs, and how these neurons interact to regulate SWR activity, remain ambiguous.

In this study, we established a computational model of the hippocampal CA3 network to explore the roles of specific interneurons in SWR generation and regulation. Our findings validate that CCKs exert an inhibitory effect on SWR events, while the activity of BCs markedly influences the occurrence of SWRs. Through this model, we anticipate that CCKs influence SWRs mainly through a direct mechanism–modulating SWR occurrence by regulating the activity of PCs. In contrast, the regulatory role of BCs is achieved by mutual inhibition within the BC population. Additionally, we noted that interactions between BCs and CCKs contribute to SWR modulation via a reciprocal inhibition mechanism. This research holds broader implications for comprehending the distinct roles of interneuron subtypes in network oscillations. By elucidating the mechanisms through which CCKs and BCs interact to modulate SWRs, our findings offer insights into the complex inhibitory control underlying memory-related oscillatory patterns. This work enriches our understanding of the neural circuitry underlying memory processing and establishes a theoretical foundation for further exploration of inhibitory control in hippocampal network dynamics.

## 2 Methods

### 2.1 Single-neuron model equations and parameters

Neurons are modeled as single-compartment, adaptive exponential integrate-and-fire (AdEx) models (Naud et al., 2008). AdEx models are characterized by the membrane potential *V* and the adaptation variable ω, which are governed by the following equations.


(1) $$\begin{array}{l} C\frac{\text{d}V}{\text{d}t}=-\left(g_{L}\left(V-V_{r}\right)-g_{L}\Delta T\exp\left(\frac{V-\vartheta }{\Delta T}\right)+I+\omega \right) \end{array}$$

(2) $$\begin{array}{l} \tau_{\omega }\frac{\text{d}\omega }{\text{d}t}=a\left(V-V_{r}\right)-\omega \end{array}$$

The AdEx model includes several key parameters: *C*, the membrane capacitance; *g*_*L*_, the leak conductance; *V*_*r*_, the reversal potential of the linear leak current, which closely approximates the resting potential; Δ*T*, the slope factor; ϑ, the intrinsic spike threshold; ω, the adaptation current; *I*, the synaptic current (Equation 3). When the membrane potential *V* exceeds the firing threshold θ, it resets to *V*_*reset*_ and remains there for a refractory period *t*_*ref*_. At each spike, the adaptation current ω increases by a fixed value *b*, representing the spike-triggered adaptation strength: ω←ω+*b*, when *V*≥θ. This additional current serves to reduce excitability following a spike, contributing to spike-frequency adaptation. The full temporal evolution of ω is then governed by the differential (Equation 2) together with this discrete increment at spike times. Additional parameters include τ_ω_, the adaptation time constant, and *a*, the adaptation coupling factor. The postsynaptic current was composed of components mediated by AMPA and GABA-A receptors and was calculated as Equation 3. The reversal potentials for excitatory and inhibitory currents were set as *E*_*exc*_ = 0 mV and *E*_*inh*_ = −70 mV , respectively.


(3) $$\begin{array}{l} I=g_{AMPA}\left(t\right)\left(V-E_{exc}\right)+g_{GABA}\left(t\right)\left(V-E_{inh}\right) \end{array}$$

The synaptic conductances *g*_*AMPA*_(*t*) and *g*_*GABA*_(*t*) evolve according to biexponential dynamics and are triggered by presynaptic spikes. Specifically, each presynaptic spike occurring at time *t*_*i*_ elicits a conductance change described by Equation 4. This ensures that synaptic currents are dynamically shaped by the timing of presynaptic activity. The Equation 4 is characterized by a peak conductance ĝ, and rise (τ_*r*_) and decay (τ_*d*_) time constants. The normalization constant $A=\exp\left(-\frac{t_{p}}{\tau_{d}}\right)-\exp\left(-\frac{t_{p}}{\tau_{r}}\right)$ was defined to ensure that the conductance reached its maximum value at *t*_*p*_ = τ_*d*_τ_*r*_/(τ_*d*_−τ_*r*_)log(τ_*d*_/τ_*r*_) ms. This approach ensured consistency in the timing of peak conductance.


(4) $$g(t)\;=\;\sum_{t_{i}<t}\hat{g}\;A(e^{-(t-t_{i})/\tau_{d}}-e^{-(t-t_{i})/\tau_{r}})$$

Table 1 summarizes the model parameters for thorny PCs, athorny PCs, BCs, and CCKs. These parameters align with experimental data and successfully reproduce voltage trajectories observed in response to a series of current pulses *in vitro* (Hunt et al., 2018; Ecker et al., 2022; Pelkey et al., 2017; Naud et al., 2008). The firing patterns of single neurons in the model are consistent with experimental observations: thorny PCs exhibit a regular-spiking firing pattern, athorny PCs display bursting activity, BCs generate fast-spiking patterns (Hunt et al., 2018), and CCKs fire at a sparser rate compared to BCs while still following a spiking pattern (Kohus et al., 2016).

**Table 1:** Model parameters and physical dimensions of thorny PCs, athorny PCs, CCKs, and BCs.

**Type**	**C**	**g_L_**	**V_r_**	***ΔT***	**ϑ**	**θ**	**V_reset_**	**t_ref_**	**τ_ω_**	**a**	**b**
Thorny PC	180.13	4.31	−75.19	4.23	−24.42	−40.40	−29.74	5.96	84.93	−0.27	206.84
Athorny PC	281.00	30.00	−65.00	2.00	−50.40	−40.40	−46.00	5.96	144.00	4.00	80.50
CCK	100.00	5.00	−57.00	2.50	−40.00	−40.00	−52.00	3.00	100.00	2.50	20.00
BC	118.52	7.51	−74.74	4.58	−57.71	−34.78	−64.99	1.15	178.58	3.05	0.91

Units: *C* (pF); *g*_*L*_ and *a* (nS); *V*_*r*_, Δ*T*, ϑ, θ, *V*_reset_ (mV); *t*_ref_ and τ_ω_ (ms); *b* (pA).

### 2.2 Network scale and connectivity

**Figure 1:**
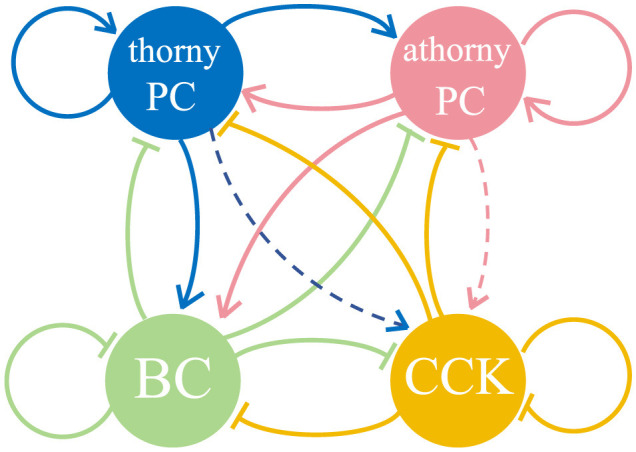
Diagram of the network and the connections among neurons, consisting of thorny pyramidal cells (thorny PCs), athorny pyramidal cells (athorny PCs), parvalbumin-expressing basket cells (BCs), and cholecystokinin-expressing interneurons (CCKs). The solid arrows represent synaptic connections between different neuronal populations, while dashed arrows indicate the absence of synaptic connections between these populations.

The network model (Figure 1) includes 8,000 heterogeneous PCs, 150 BCs, and 100 CCKs, representing the CA3 region within a 600-μm-thick slice of the mouse hippocampus (English et al., 2014; Donoso et al., 2018; Ecker et al., 2022), where SWR activity has been observed experimentally (Hájos et al., 2013; Schlingloff et al., 2014; Gan et al., 2017). Among the PCs, 15% are designated as athorny PCs, amounting to 1,200 neurons, while the remaining 6800 are thorny PCs. This proportion aligns with prior experimental findings that report athorny PCs comprising 10%-20% of the total PC population (Hunt et al., 2018). To model spatial selectivity, 50% of both thorny PCs and athorny PCs were randomly selected as place cells, resulting in 3400 thorny place cells and 600 athorny place cells. Place fields were assigned randomly, allowing for overlaps (Ecker et al., 2022). These place cells generated spike trains following inhomogeneous Poisson processes with time-varying firing rates, λ(*t*), determined by place cell-like tuning curves. The tuning curves were Gaussian-shaped, centered at random positions, with a standard deviation (σ) designed to cover 10% of the 3-meters-long linear track. The firing rate at the edge of the position field is 10% of the maximum 20 Hz (Dragoi and Buzsáki, 2006). Spike timing was modulated by background theta oscillations (*f*_θ_ = 7 Hz) and phase precession (O'Keefe and Recce, 1993). Spiking events for place cells were generated using an accept-reject sampling approach based on their tuning curves, ensuring that firing was modulated by both spatial and temporal dynamics. The firing rate of the *i*-th place cell was expressed as:


(5) $$\begin{array}{l} \tau_{i}\left(x\right)=\exp\left(\frac{-{\left(x-m_{i}^{PF}\right)}^{2}}{2\sigma^{2}}\right) \end{array}$$

(6) $$\begin{array}{l} \lambda_{i}\left(t\right)=\hat{\lambda }\times \tau_{i}\left(x\left(t\right)\right)\times \cos\left(2\pi f_{\theta }t+\frac{\pi }{l^{PF}}\left(x\left(t\right)-s_{i}^{PF}\right)\right) \end{array}$$

where *x*(*t*) represents the position of the animal, and $m_{i}^{PF}$, *l*^*PF*^, and $s_{i}^{PF}$ denote the center, length, and start position of the place field, respectively (*l*^*PF*^ = 0.3 m). The parameter $\hat{\lambda }$ was set to 20 Hz as the maximum firing rate within the field. Non-place cells maintained a low, spatially non-specific activity, firing at a average rate of λ = 0.1 Hz.

The spike trains generated by thorny PCs and athorny PCs were utilized as inputs for spike-timing-dependent plasticity (STDP) rules, resulting in two distinct synaptic weight matrices for recurrent excitatory connections. This approach was selected because the synaptic connections formed by pyramidal neurons, including both thorny PCs and athorny PCs, are crucial for memory-related functions. By applying STDP to these connections, we dynamically adjusted the synaptic weights based on the precise spike timing of thorny PCs and athorny PCs, thereby simulating the synaptic plasticity involved in memory formation and maintenance. Subsequently, synaptic connections among thorny PCs and athorny PCs were established according to their respective learned weight matrices. For all other neuronal populations, random connectivity was implemented as the network topology, reflecting the distinct connectivity patterns of these populations, which is consistent with their role in maintaining network synchrony.

Recent studies have revealed an asymmetric connectivity pattern between thorny PCs and athorny PCs, where athorny PCs receive strong excitatory input from thorny PCs, while thorny PCs receive comparatively weaker input from athorny PCs (Sammons et al., 2024; Marissal et al., 2012). To incorporate this asymmetry into the model, we adjusted the connectivity accordingly. The 10% connectivity probability from PCs to BCs aligns with the classical view that the CA3 region represents a highly interconnected network (Lisman, 1999; Ecker et al., 2022). This high degree of connectivity facilitates the robust generation of SWRs and supports complex network dynamics. Consistent with prior findings, the connection probability from BCs to other neuron groups, as well as BC self-connections, was set to 25% (Schlingloff et al., 2014).

For CCKs, extensive research suggests that these neurons receive minimal glutamatergic input, with most of their excitatory drive originating from subcortical regions and being influenced by external modulatory systems (Freund, 2003; Bezaire et al., 2016). Based on this evidence, no synaptic connections were established between CCKs and the two types of PCs in our model. Instead, an external current was applied to CCKs. Additionally, studies indicate that the coupling strength within the CCK population is weaker than that among BCs (Freund, 2003; Booker and Vida, 2018), and the connection probabilities between CCKs and other neuron types are also lower (Freund and Katona, 2007). These findings were incorporated into the model by appropriately setting the relevant connectivity parameters. Table 2 summarizes the parameters of synaptic connections between neuronal populations. The majority of these synaptic parameters were adapted from previous modeling work (Ecker et al., 2022), with parameter selection being informed by experimental data from earlier studies (Geiger et al., 1997; Bartos et al., 2002; Lee et al., 2014; Vyleta et al., 2016; Guzman et al., 2016). These parameters were further refined using an evolutionary algorithm implemented in BluePyOpt with a custom evaluator. For synaptic connections involving athorny PCs, direct experimental measurements are currently unavailable; thus, we applied the same synaptic parameter values as thorny PCs.

**Table 2:** The synaptic parameters and physical dimensions for the four types of neuronal connections.

**Connection**	**ĝ**	**τ_*r*_**	**τ_d_**	**t_d_**	**p_conn_**
Thorny PC → Thorny PC	0.1-6.3	1.3	9.5	2.2	-
Thorny PC → BC	0.85	1.0	4.1	0.9	0.1
Thorny PC → Athorny PC	1.0	1.3	9.5	2.2	0.11
BC → Thorny PC	0.65	0.3	3.3	1.1	0.25
BC → Athorny PC	0.65	0.3	3.3	1.1	0.25
BC → BC	5.0	0.25	1.2	0.6	0.25
Athorny PC → Thorny PC	3.0	1.3	9.5	2.2	0.04
Athorny PC → Athorny PC	0.1-6.1	1.3	9.5	2.2	-
Athorny PC → BC	0.85	1.0	4.1	0.9	0.1
CCK → Thorny PC	0.65	0.3	3.3	1.1	0.12
CCK → Athorny PC	0.65	0.3	3.3	1.1	0.12
CCK → CCK	5.0	0.25	1.2	0.6	0.2
CCK → BC	5.0	0.25	1.2	0.6	0.12
GC → Thorny PC	19.15	0.65	5.4	-	-
EC → Athorny PC	19.15	0.65	5.4	-	-

Units: ĝ (nS); τ_*r*_, τ_*d*_, and *t*_*d*_ (synaptic delay) (ms); *p*_conn_ (connection probability) is unitless. GC represents the granule cells in the dentate gyrus, and EC represents the entorhinal cortex.

### 2.3 Spike-timing-dependent plasticity (STDP) rule

In our model, the synaptic weights for thorny PCs and athorny PCs were initialized at 0.1 nS and adjusted based on the spike-timing-dependent plasticity (STDP) rule as follows (Mishra et al., 2016):


(7) $$\begin{array}{l} \Delta w_{+}=A_{+}\exp\left(-\frac{\Delta t}{\tau_{+}}\right)att_{post}\text{if}t_{pre}<t_{post}, \end{array}$$

(8) $$\begin{array}{l} \Delta w_{-}=A_{-}\exp\left(\frac{\Delta t}{\tau_{-}}\right)att_{pre}\text{if}t_{pre}>t_{post}, \end{array}$$

where Δ*t* = *t*_*post*_−*t*_*pre*_ is the time difference between action potentials, to achieve a symmetric STDP curve, *A*_±_ was set to 80 pA. The weight update followed an exponential decay, governed by the time constants τ_±_ = 62.5 ms, The synaptic weights were capped at a maximum value of ω_*max*_ = 20 nS to prevent unbounded growth.

### 2.4 Stimulus simulation

We developed a small-scale neural circuit model of the hippocampal CA3 region, consisting of excitatory neurons–thorny PCs and athorny PCs, as well as two types of interneurons: BCs and CCKs. To simulate the excitatory inputs received by these neurons *in vivo*, we applied specific types of stimuli to each neuronal population based on their known physiological properties. Thorny PCs received Poisson-distributed spike train input with a mean frequency of 16 Hz. This input was designed to mimic the robust excitation from mossy fibers, which provide strong and frequent synaptic drive to thorny PCs (Aussel et al., 2018; Jung and McNaughton, 1993; Cerasti and Treves, 2010). In contrast, athorny PCs received Poisson-distributed spike train input with a mean frequency of 2.7 Hz. This weaker and less frequent input reflects the cortical and other brain region afferents that primarily drive athorny PCs. The lower intensity of stimulation received by athorny PCs, compared to thorny PCs, is attributed to their minimal excitatory input from mossy fibers (Hunt et al., 2018). CCKs were stimulated by a constant external current of 0.2 nA. This choice was based on the fact that CCKs receive minimal glutamatergic input and are primarily modulated by external regulatory systems (Freund, 2003; Booker and Vida, 2018). BCs did not receive additional external input, as their primary excitatory drive comes from glutamatergic input from PCs. Instead, their activity was driven by synaptic inputs from thorny PCs and athorny PCs within the network (Freund and Katona, 2007). Under these stimulus conditions, our model produced spontaneous SWR activity, replicating a fundamental oscillatory phenomenon observed in the CA3 region.

### 2.5 Detection of SWR

**Figure 2:**
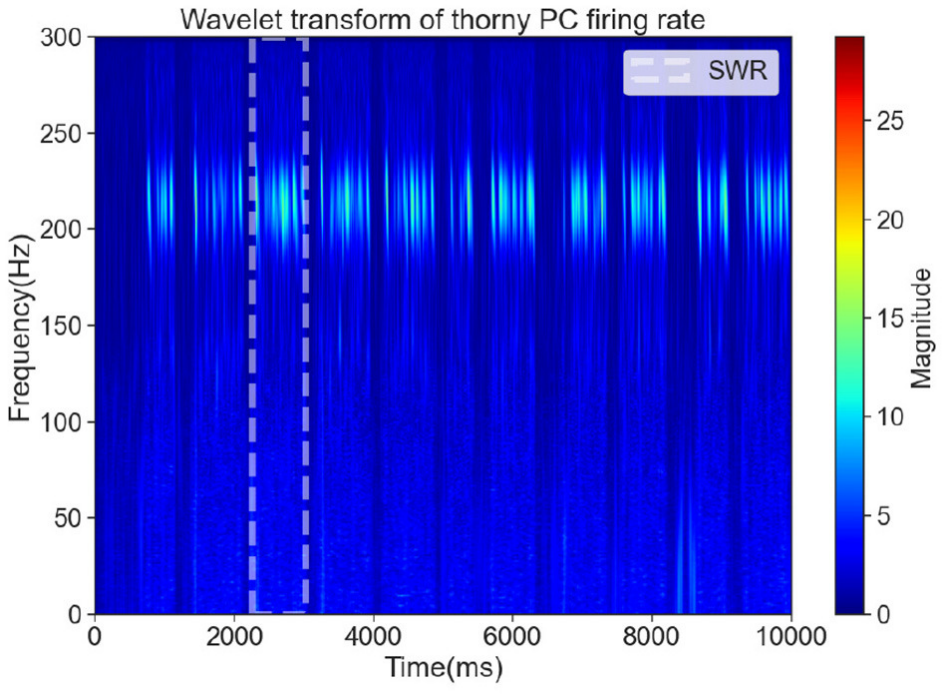
The time-frequency representation of a significant SWR event, marked by a white dashed box.

Although ripples are most prominently observed in the CA1 region, the CA3 region plays a critical role in the generation of sharp waves, which are the primary drivers of SWRs. Experimental studies have shown that CA3 PCs exhibit synchronized bursting activity during sharp waves, which propagate to CA1 and give rise to high-frequency ripple oscillations (Buzsáki, 1986). Therefore, analyzing the activity of CA3 PCs provides valuable insights into the initiation and modulation of SWRs (Raus Balind et al., 2019; Ecker et al., 2022; Bazelot et al., 2016). In our CA3 network model, we focus on the dynamics of thorny PCs, which are the primary contributors to sharp wave generation, and use time-frequency analysis to detect SWR-related activity. To detect SWRs in the CA3 network, we performed time-frequency analysis on the spike trains of thorny PCs. The spike trains were analyzed using the Wavelet Transform to generate time-frequency representations. In these representations, sharp waves were identified as low-frequency (1–30 Hz) high-amplitude events, while ripples were identified as high-frequency (120–250 Hz) oscillations (Hofer et al., 2015). SWR events were defined as the co-occurrence of sharp waves and ripples within a specified time window. The white dashed box in Figure 2 highlights a significant period during which both sharp waves and ripple frequencies were detected, consistent with the characteristic features of SWRs observed in experimental studies (Buzsáki, 1983; Green and Arduini, 1954).

### 2.6 Metrics for assessing network dynamics

To analyze the dynamical states of the network, several computational metrics were defined.

Normalized firing intensity: the normalized firing intensity was calculated over the time window [3,500, 6,500] ms, spanning 1500 ms before and after the application of current at 5000 ms. With a time bin of 20 ms, the normalized firing intensity was defined as the number of spikes within each time bin divided by the maximum spike count observed across all bins. This metric enables direct comparisons of the firing activity among different neuron types on a consistent scale, facilitating the evaluation of firing intensity, relative changes, and activation levels. It provides insights into the firing behavior of various neuron types during and around SWRs.

High-Activity segments: high-activity segments were identified based on thorny PCs exhibiting firing rates above a threshold of 2 Hz for a sustained period of at least 260 ms (with a time bin size of 20 ms) (Ecker et al., 2022).

SWR power: Firstly, for high-activity segments that passed a statistical significance test, the proportion of power in the 120-250 Hz band relative to the total power was computed; Secondly, the average of these proportions across all high-activity segments was taken as the Ripple Power, representing the percentage contribution of SWR power to the overall neural activity. This metric evaluates the intensity of SWRs in the context of overall network activity.

Average SWR frequency: Firstly, the ripple band (150-220 Hz) of the power spectrum from high-activity segments was subjected to a significance test, retaining only those segments that passed the Fisher's g-statistic (Fisher, 1929). Secondly, for each retained segment, the frequency with the maximum power in the ripple band was extracted. Thirdly, the average of these peak frequencies was calculated to obtain the Average SWR Frequency, which reflects the dominant frequency components of SWRs in the network and provides key frequency characteristics.

Synchrony index: Firstly, for each detected SWR event, spike times of pyramidal cells within the corresponding time window were extracted. Secondly, these spikes were binned using non-overlapping 5 ms time bins to compute the population firing rate over time. Thirdly, the variance of the binned firing rate was calculated for each SWR period, serving as an index of population synchrony–the higher the variance, the greater the temporal concentration of spikes, and thus, the higher the synchrony. Finally, a synchrony index measure was obtained by averaging the firing rate variances across all SWR events.

## 3 Results

### 3.1 Inhibitory effect of CCK on SWR generation

Previous studies have demonstrated that the activity of interneuron CCKs in the hippocampal CA3 region typically declines before the onset of SWRs, suggesting that CCKs contribute to shaping the inhibitory tone preceding SWR events (Vancura et al., 2023). To explore the specific regulatory impact of CCKs on SWRs, we carried out a series of computational simulations in a detailed hippocampal CA3 network model.

**Figure 3:**
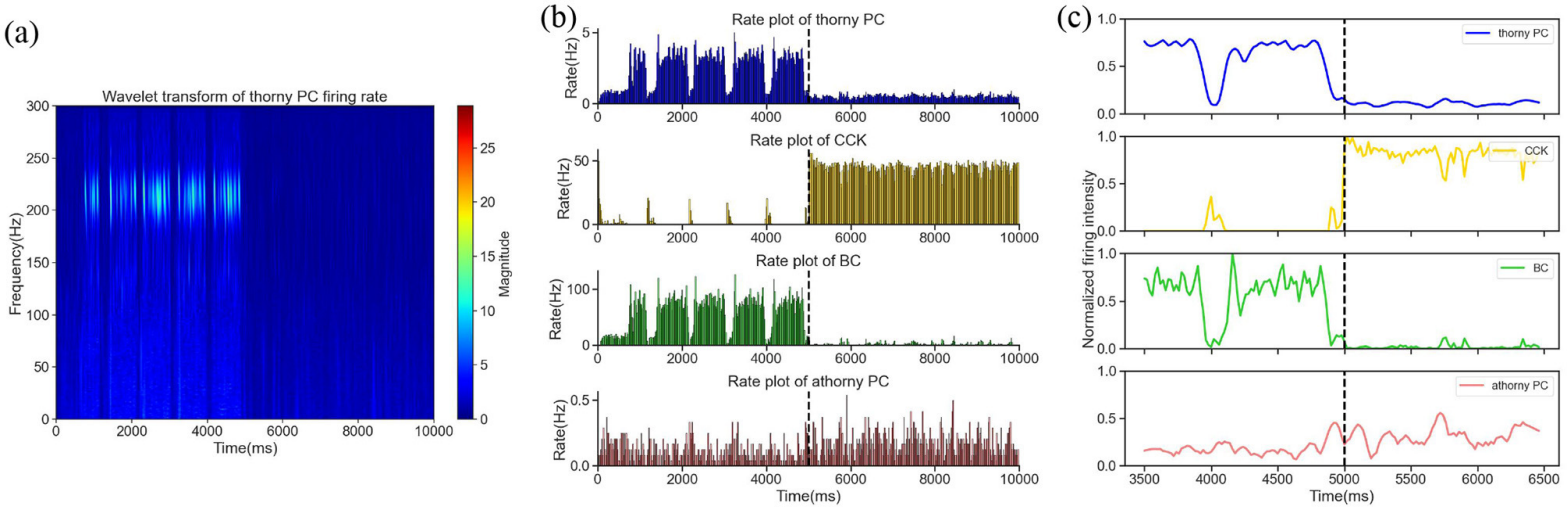
**(a)** The time-frequency representation within the [0, 10,000] ms time window. **(b)** The firing rates of four neuron groups–thorny PCs, CCKs, BCs and athorny PCs–throughout the entire simulation period. The dashed line at 5,000 ms marks the initiation of the increased current stimulation. **(c)** Smoothed curves of the normalized firing activity of the four neuron groups during a 1,500 ms window before and after the current application, spanning [3,500, 6,500] ms. The dashed line denotes the onset of the current application at 5,000 ms.

The simulation duration was set at 10,000 ms and divided into two distinct phases. During the first half of the simulation ([0, 5,000] ms), interneuron CCKs were exposed to a 0.2 nA excitatory current input, which mimicked the influence of an external excitatory regulatory system. This input was intended to simulate the physiological conditions under which CCKs might become activated. The time-frequency representation is shown in Figure 3a, within this period, we witnessed a combination of synchronous and sparse firing patterns in the excitatory thorny PCs, corresponding to both SWR and non-SWR states in the CA3 network. Notably, during SWR events, which were identified by the synchronous firing of PCs, CCKs demonstrated minimal firing, while BCs exhibited high firing rates, as shown in Figure 3b, indicating an anti-phase relationship between BC and CCK activity, consistent with previous experimental observations (Kohus et al., 2016). In the second half of the simulation ([5,000, 10,000] ms), an additional 0.3 nA excitatory current was applied to the CCKs, besides the initial 0.2 nA input. This augmented the activity of the CCKs and maintained them in a state of sustained high activity, as illustrated in Figure 3c. During this period, a significant reduction in the firing rates of both thorny PCs and BCs was observed, reflecting a shift in network dynamics. In the initial 5000 ms, SWRs emerged as anticipated, but following the increased CCK stimulation in the second phase, SWRs were absent, suggesting that the heightened activity of CCKs exerted a potent inhibitory influence on the network, particularly on the thorny PCs. This inhibition resulted in the suppression of SWR occurrence. To further examine whether this modulation exhibits a graded pattern, we additionally tested excitatory input levels of 0.3 nA, 0.4 nA and 0.6 nA. The results showed a progressive reduction in SWR frequency as CCK activation increased, suggesting that the inhibitory influence of CCKs on network dynamics is activity-dependent and continuous. This indicates that CCKs can dynamically regulate SWR generation across different levels of excitatory drive.

In summary, consistent with prior experimental findings, our simulation results support the experimental observation that CCKs exert an inhibitory effect on SWR generation in the hippocampal CA3 network. These findings contribute to a deeper understanding of the role of specific interneuron populations in regulating memory-related oscillatory patterns. Moreover, the results provide valuable insights into how changes in CCK activity might influence hippocampal network states, with implications for learning and memory processes.

### 3.2 CCKs suppress SWR generation via direct modulation of PC excitability

The hippocampus comprises a heterogeneous population of interneurons, each exhibiting unique biophysical characteristics and connectivity profiles. This heterogeneity complicates the design of experiments aimed at elucidating the specific mechanisms by which these interneurons contribute to network oscillations. Among these interneurons, the functional role of CCKs in SWR activity remains incompletely understood. Since the synchronized firing of PCs is fundamental to SWR generation, we investigated the inhibitory interactions between CCKs and PCs to clarify how CCKs modulate this process. To achieve this, we utilized a mathematical modeling approach to analyze the underlying inhibitory mechanisms and to identify the principal neural pathway through which CCKs regulate SWR dynamics.

**Figure 4:**
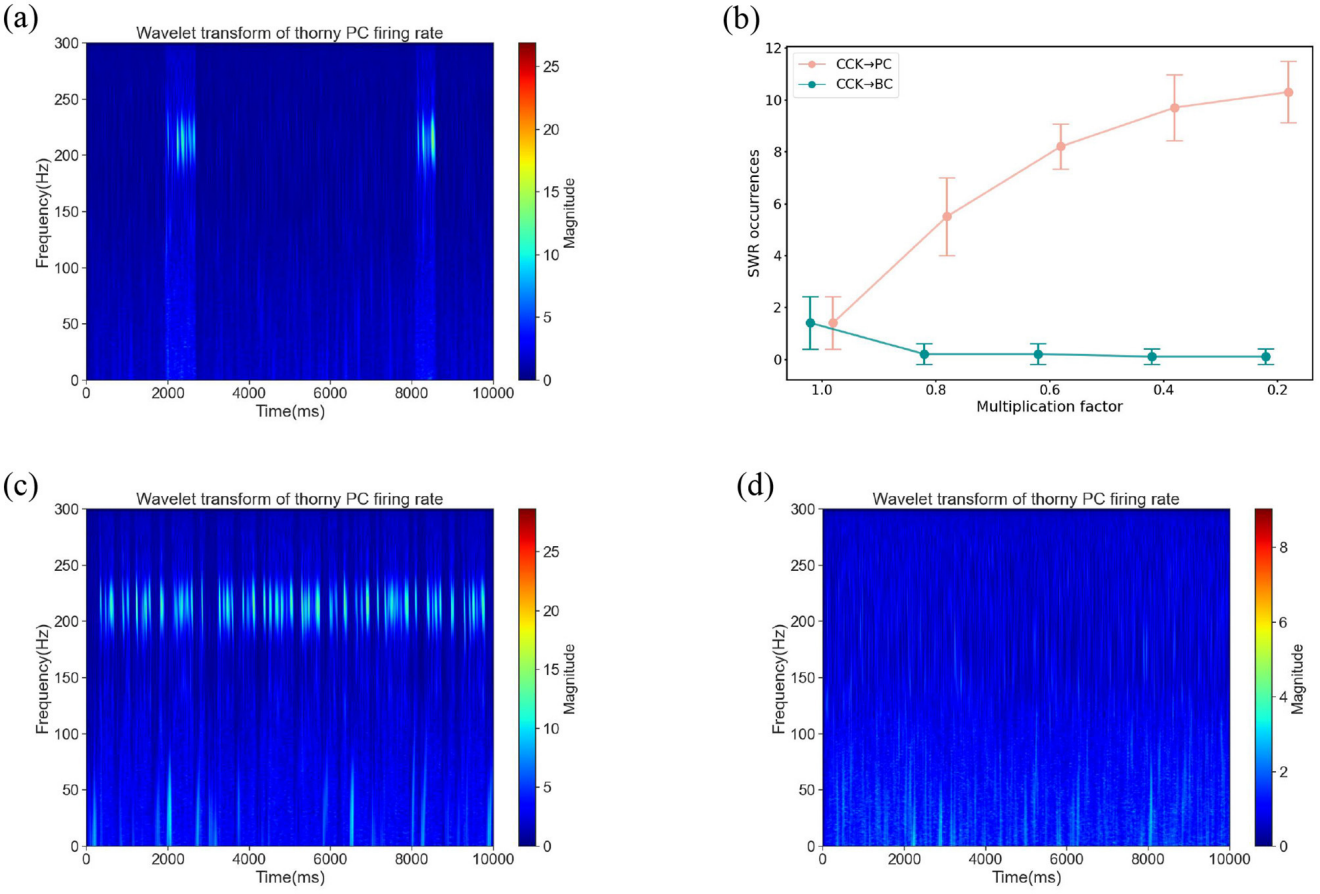
**(a)** The time-frequency representation under a background current of 0.4 nA applied to CCKs. At this current level, CCKs exhibit heightened excitability. **(b)** SWR occurrences in the network after scaling the synaptic weight of CCKs → PCs and CCKs → BCs connections by a series of multiplicative factors [1.0, 0.8, 0.6, 0.4, 0.2], respectively. Error bars represent the standard deviation, with ten simulations performed under different random seeds for each weight factor. **(c)** The time-frequency representation subsequent to the removal of synaptic connections from CCKs to PCs (CCKs → thorny PCs and CCKs → athorny PCs) under a background current of 0.4 nA applied to CCKs. **(d)** The time-frequency representation subsequent to the removal of synaptic connections from CCKs to BCs (CCKs → BCs) under a background current of 0.4 nA applied to CCKs.

Our network model disclosed two potential routes through which CCKs could modulate the synchronous firing of PCs: a direct CCK → PC synaptic route and an indirect CCK → BC → PC route, where CCKs act upon BCs, which in turn affect PCs. We initially simulated the activation of CCKs by applying a depolarizing excitatory current to the CCKs. Under this circumstance, the time-frequency representation is shown in Figure 4a, this stimulation largely suppressed the SWR events, suggesting that the enhanced activity of CCKs disrupts SWR generation. To investigate the specific mechanism underlying this effect, we performed a series of systematic simulations. In these simulations, we progressively reduced the synaptic conductances of the CCK → PC and CCK → BC connections by scaling them with factors of 1.0, 0.8, 0.6, 0.4, and 0.2. For each condition, we quantified the number of SWR events. As illustrated in Figure 4b, attenuating the CCK → PC connections led to a gradual increase in SWR occurrences, suggesting that CCKs primarily inhibit SWRs through direct interactions with PCs. Conversely, weakening the CCK → BC connections did not result in a significant recovery of SWRs; the network remained largely inactive, with SWR activity persisting at a low level across all attenuation levels. To further validate these findings, we conducted two additional control simulations. When the CCK → PC synaptic connections were entirely eliminated, SWRs reappeared robustly (Figure 4c), thereby reinforcing the conclusion that this direct inhibitory pathway plays a critical role in suppressing SWR generation. In contrast, removing the CCK → BC pathway had no substantial effect on SWR reappearance (Figure 4d), further supporting the limited contribution of the indirect pathway in this context. Collectively, these findings uphold the hypothesis that CCKs inhibit SWR generation through a direct mechanism. Specifically, when CCKs are activated, they exert a direct inhibitory effect on thorny PCs via synaptic connections. This direct modulation diminishes the excitability of the thorny PCs, thereby suppressing the synchrony of their population activity and, consequently, impeding the generation of SWRs.

These results significantly enhance our comprehension of the role of CCKs in hippocampal network dynamics, illuminating the specific mechanisms through which they regulate neural oscillatory activity. Our discoveries reveal that CCKs exert their modulatory influences predominantly via direct interactions with pyramidal cells, rather than indirectly through other inhibitory circuits. This emphasizes their crucial contribution to shaping sharp-wave ripple events and broader network oscillatory behavior, offering novel insights into the functional significance of interneuron-specific modulation within the hippocampus.

### 3.3 The impact of BC activity on SWR generation and its regulatory mechanism

The SWR oscillation pattern in the hippocampal CA3 region is intricately regulated by the activity of various types of inhibitory interneurons. In addition to CCKs, extensive research has demonstrated that BCs also play a critical role in modulating the dynamics of SWR events. For instance, experiments conducted in 2014 showed that optogenetic silencing of BCs prevented the occurrence of SWRs, while optogenetic activation of BCs induced full SWR sequences along with excitatory postsynaptic current (EPSC) events (Schlingloff et al., 2014). To explore the role of BCs in SWR generation, we conducted a series of computational simulations to investigate how changes in BC activity impact SWR dynamics.

**Figure 5:**
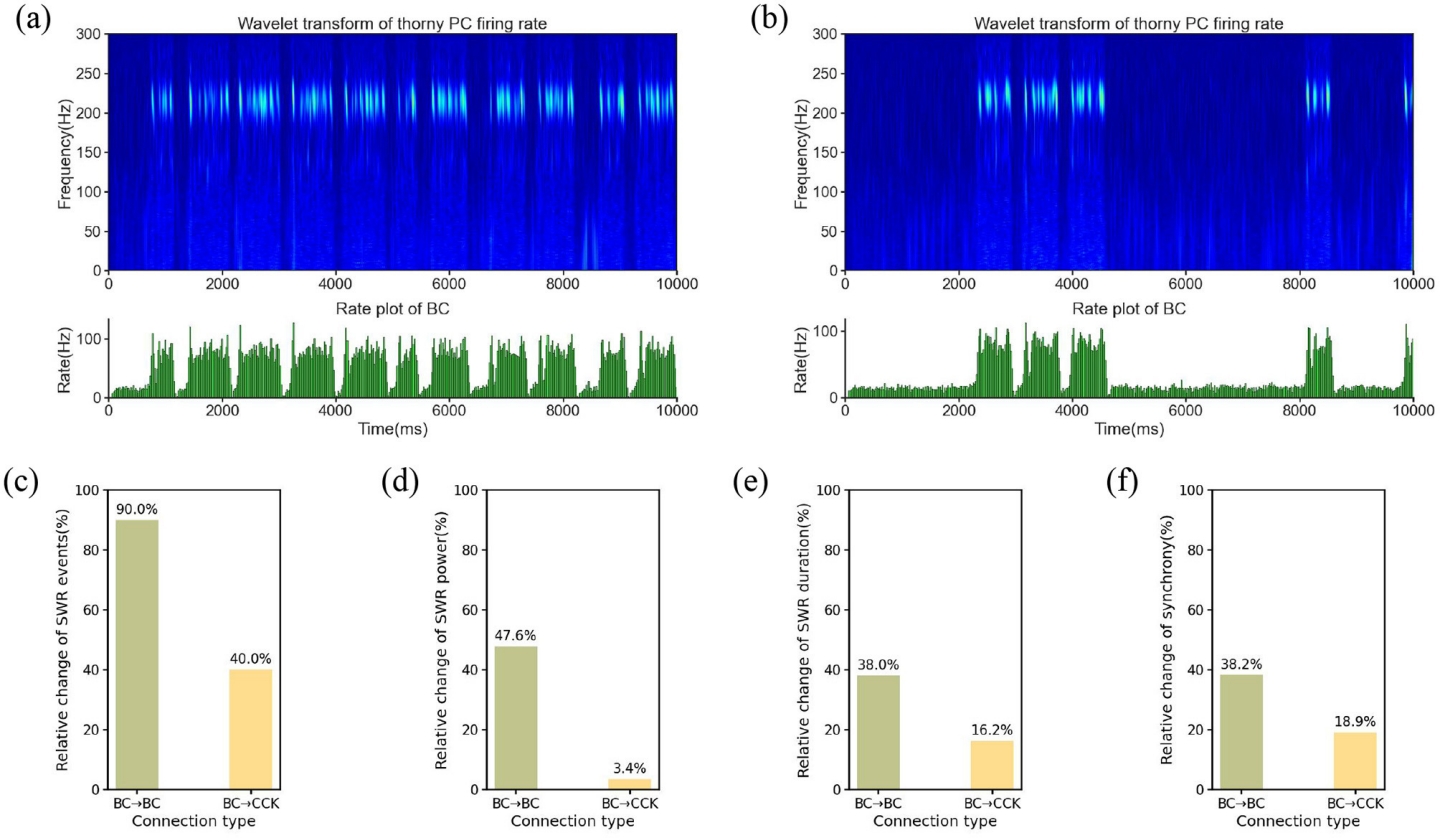
**(a)** Time-frequency representation and average firing rate of the BC population under normal BC activity. **(b)** Time-frequency representation and average firing rate of the BC population under suppressed BC activity. **(c–f)** Relative changes in SWR characteristics were evaluated under modulation of BC → BC and BC → CCK synaptic conductances, which were systematically scaled by factors [0.8, 0.9, 1.0, 1.1, 1.2]. **(c)** Relative change in SWR event count. **(d)** Relative change in SWR power. **(e)** Relative change in SWR duration. **(f)** Relative change in synchrony.

In the first simulation, BCs maintained their normal activity levels, reflecting the standard network state where SWRs alternated with non-SWR states, consistent with the typical dynamics of the hippocampal CA3 region. During this period, the network remained stable, and BCs exhibited regular firing patterns, contributing to the overall balance of network activity. To visualize the temporal dynamics of SWRs under this condition, we constructed a time-frequency representation. The time-frequency representation revealed a robust presence of SWR events, indicating the normal occurrence of these oscillatory patterns when BCs were active (Figure 5a). In the second simulation, we modulated BCs activity by reducing their intrinsic excitability. Specifically, we decreased the leak conductance of BCs to 50% of its original value. This manipulation effectively reduced BC firing activity, as the lowered leak conductance resulted in a hyperpolarized resting membrane potential and decreased responsiveness to synaptic inputs. The time-frequency representation during this simulation demonstrated a significant reduction in the occurrence of SWR events compared to the baseline condition, as shown in Figure 5b. This observation suggests that the suppression of BCs activity disrupts the network dynamics necessary for SWR generation.

The contrasting time-frequency representation patterns between the two conditions highlight the critical role of BCs in regulating SWR dynamics. The reduction in SWR occurrence during BC suppression implies that BCs are essential for the initiation and maintenance of these oscillatory events. By modulating the activity of PCs and other network elements, BCs serve as key regulators of SWR generation in the hippocampal CA3 region. This finding underscores the importance of BCs in maintaining the balance of network dynamics and suggests that alterations in BC activity could have significant implications for hippocampal function, particularly in processes such as memory consolidation that rely on SWRs.

Current experimental studies have revealed two primary mechanisms through which BCs regulate SWR dynamics: one is mutual inhibition between BCs (Wang and Buzsáki, 1996; Brunel and Wang, 2003; Taxidis et al., 2012), and the other is a disinhibition mechanism, where BCs suppress the activity of another anti-SWR neuron population (i.e., CCK), and the anti-SWR neuron population subsequently affects SWR generation (Evangelista et al., 2020). To determine which of these mechanisms plays a more significant role in regulating SWR dynamics, we conducted the following investigations.

We systematically modified the synaptic weights of two key connections: BC → BC and BC → CCK. These synaptic weights were adjusted by multiplying them with a series of scaling factors, precisely [0.8, 0.9, 1.0, 1.1, 1.2]. Subsequently, we systematically extracted and analyzed several quantitative features of SWRs under each condition, including the number of SWR events, SWR power, SWR duration, and synchrony. As shown in Figures 5c–f, modulating the BC → BC synaptic strength resulted in more substantial changes across all four SWR features compared to modulating the BC → CCK connection. Specifically, as the BC → BC synaptic weight increased, noticeable alterations were observed in SWR power, duration, synchrony, and the number of events, with the number of SWR events showing the largest relative change. In contrast, adjusting the BC → CCK synaptic weight had only a minimal impact on these characteristics, with slight fluctuations observed across all metrics. Quantitatively, modulating the BC → BC synapse induced a 90.0% relative change in event count, a 47.6% relative change in SWR power, a 38.0% relative change in average duration, and a 38.2% relative change in synchrony, whereas the corresponding changes from BC → CCK modulation were 40.0%, 3.4%, 16.2%, and 18.9%, respectively. These findings distinctly indicate that adjustments to the BC → BC synapse exert a significantly more pronounced influence on both the intensity and spatiotemporal dynamics of SWRs compared to modulations of the BC → CCK connection.

Based on these results, we propose that the primary mechanism by which BCs regulate SWRs is through mutual inhibition between BCs. The importance of this inhibitory interaction highlights the critical role of local inhibitory circuits in regulating large-scale brain oscillations, such as SWRs, which are essential for processes like memory consolidation and synaptic plasticity. By understanding the contribution of BC-BC mutual inhibition, we gain deeper insights into the fundamental principles governing the dynamics of hippocampal network activity and its role in cognitive functions.

### 3.4 The role of mutual inhibition between BCs and CCKs in regulating SWR dynamics

**Figure 6:**
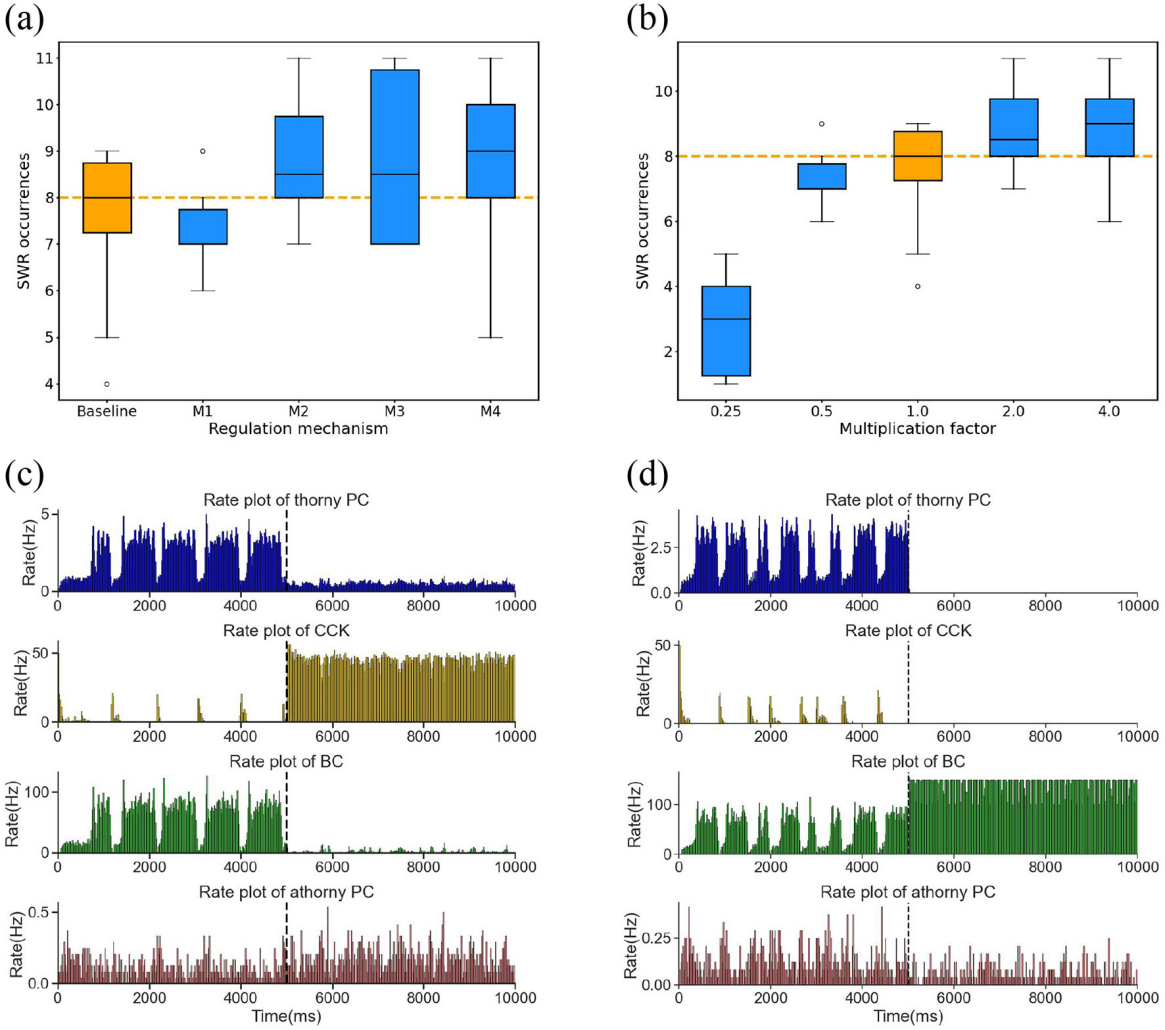
**(a)** Boxplot of the number of SWR occurrences under the baseline condition and four regulatory mechanisms correspond to a series of adjustments to the synaptic weights of BCs → CCKs and CCKs → BCs. Each condition incorporates simulations with ten distinct random seeds. Baseline represents SWR occurrences under normal synaptic weights. The orange dashed line indicates the median of the baseline. The mechanisms are defined as follows: M1: ω(BCs → CCKs) × 0.5, ω(CCKs → BCs) × 0.5; M2: ω(BCs → CCKs) × 2.0, ω(CCKs → BCs) × 2.0; M3: ω(BCs → CCKs) × 0.5, ω(CCKs → BCs) × 2.0; M4: ω(BCs → CCKs) × 2.0, ω(CCKs → BCs) × 0.5. **(b)** Boxplot of the number of SWR occurrences subsequent to the symmetrical multiplication of the synaptic weights of BCs → CCKs and CCKs → BCs by a series of multiplication factors [0.25, 0.5, 1.0, 2.0, 4.0]. The orange dashed line indicates the median of the baseline. **(c)** Firing rates of the four neuronal populations (thorny PCs, athorny PCs, CCKs, and BCs) before and after applying external current to CCKs. The dashed line indicates the onset of current injection. **(d)** Firing rates of the four neuronal populations (thorny PCs, athorny PCs, CCKs, and BCs) before and after applying external current to BCs. The dashed line indicates the onset of current injection.

Previously, we investigated the individual regulatory mechanisms of CCK and BC interneurons on SWR generation. However, the interaction between these two types of interneurons is also crucial for the stability of SWRs. Despite this, the specific nature of their interaction and its impact on SWR regulation remains unclear. To specifically examine the role of mutual inhibition between BC and CCK interneurons, we maintained the activity levels of both populations at their physiological baseline and manipulated only the synaptic weights of the BC → CCK and CCK → BC pathways. This allowed us to selectively modulate the strength of their reciprocal interaction without altering their intrinsic excitability. We examined the influence of these adjustments on the frequency of SWR occurrences over a 10,000 ms simulation period. Four regulatory mechanisms were tested: M1, M2, M3, and M4, each corresponding to different degrees of symmetric or asymmetric modulation of the two synaptic weights, the result as illustrated in the Figure 6a.

We concluded that the interaction between BC and CCK exerts a more symmetrical impact on regulating SWR, namely, a mutual inhibition mechanism. When the synaptic weights of both pathways were symmetrically reduced to half of their original values (corresponding to M1), the occurrence of SWRs decreased. This reduction in mutual inhibition weakened the overall control of BC and CCK over PC activity, leading to diminished synchronization in the network. Consequently, this attenuation of mutual inhibition made it difficult for the network to achieve the necessary synchrony for high-frequency SWR oscillations, thus decreasing the frequency of SWR events. Conversely, when the synaptic weights of both pathways were doubled (corresponding to M2), the mutual inhibitory regulation between BC and CCK was more effective, resulting in greater synchronization of network activity and an increase in SWR occurrences. In contrast to symmetric modulation, asymmetric modulation of synaptic weights (corresponding to M3 and M4 mechanisms) disrupted the balance of mutual inhibition between BCs and CCKs. Specifically, when the synaptic weight of one pathway was reduced to half its original value while the other was doubled, the mutual inhibition between BCs and CCKs became fundamentally imbalanced. This imbalance destabilized the inhibitory control within the network, leading to increased network excitability. Consequently, the frequency of SWR events increased compared to baseline conditions.

To further confirm the mutual inhibition between BC and CCK, we applied symmetric modulation to the synaptic weights of the BC → CCK and CCK → BC pathways, by scaling both weights using a series of multiplication factors [0.25, 0.5, 2.0, 4.0], as shown in the Figure 6b. We observed that as the scaling factors increased, the strength of the interaction between BC and CCK also increased, leading to stronger regulation of SWRs. Additionally, we conducted two simulations. When excitatory current was applied to CCK neurons, their firing rate increased, and simultaneously, the firing rate of BC neurons decreased, indicating that CCK has an inhibitory effect on BC neurons, as shown in the Figure 6c. On the other hand, when excitatory current was applied to BC neurons, their activity increased, and CCK firing decreased, suggesting that BC neurons inhibit CCK activity, as depicted in the Figure 6d. These results clearly indicate that BC and CCK interact through mutual inhibition and their interaction exerts a symmetric effect on SWR regulation.

In summary, we conclude that BC and CCK regulate SWRs through a mutual inhibition mechanism. This mutual inhibition is critical for maintaining network stability and dynamic balance, which are essential for proper SWR regulation and cognitive functions such as memory consolidation.

## 4 Conclusion and discussion

This study has illuminated the crucial roles of interneurons, specifically CCKs and BCs, in governing SWR within the hippocampal CA3 network. By employing a computational modeling approach, we manifested that interneuron CCKs suppress SWR generation via a direct mechanism entailing the modulation of PCs activity. In contrast, interneuron BCs exert influence on SWRs mainly through mutual inhibition among BCs, which is indispensable for attaining the stability and synchronization requisite for SWR oscillations. Furthermore, we identified a mutual inhibitory interaction between CCKs and BCs, which exerts a relatively symmetrical effect on SWR regulation. This interaction highlights the complex interplay among these interneuron populations and emphasizes their collective influence on network dynamics. These findings stress the significance of specific inhibitory neuronal subtypes and their interactions in maintaining the equilibrium of excitatory and inhibitory signaling, which is crucial for proper hippocampal function, including oscillatory activity and memory consolidation.

Our model results are consistent with key experimental observations reported by Vancura et al. (2023). Specifically, we found that CCK+ interneurons exhibit decreased activity prior to SWRs and increased activity following SWRs, which closely mirrors the temporal dynamics observed in the experimental data. Similarly, BCs in our model show elevated activity during SWRs and a reduction in activity during non-SWR periods, consistent with the experimental findings. These observations underscore the biological relevance of our CA3 network model in capturing key aspects of hippocampal interneuron behavior. However, one notable discrepancy between our simulations and experimental data is the duration of the SWRs. In our model, SWRs appear to be somewhat prolonged compared to those observed in experimental recordings. This difference may arise from the simplifications inherent in the model or from the specific parameter choices made in our simulations. Future work could refine these parameters to better align the model dynamics with experimental observations, enhancing the model's fidelity in simulating the full temporal characteristics of SWRs. Despite this, the overall pattern of interneuron activity in our model remain in strong agreement with experimental data, supporting the biological validity of our approach. Another limitation of the current study lies in its focus on the local circuit mechanisms by which interneurons (CCKs and BCs) regulate SWR generation. While our model successfully identifies the roles and interactions of these cell types in modulating SWR dynamics, it does not extend to exploring the functional consequences of these dynamics–such as their roles in sequence replay, information encoding, or memory consolidation. Since SWRs are critically involved in these higher-order processes, future work could build upon our findings to investigate how interneuron-mediated modulation of SWRs affects the fidelity or content of neural replay and memory-related functions. Incorporating such functional analyses would provide a more comprehensive understanding of the behavioral significance of interneuron contributions to hippocampal network activity. In addition, significant changes in neuronal composition may under certain conditions lead to altered network dynamics and even pathological states such as hyperexcitability or desynchronization. For instance, reduced heterogeneity among inhibitory interneurons has been linked to increased risk of pathological synchronization (Rich et al., 2022). While our study focuses on a physiologically normal CA3 network, exploring these effects represents an important direction for future work.

Our discoveries also possess broader implications for comprehending synaptic plasticity. The mutual inhibitory interactions between CCK and BC interneurons imply that the strength and plasticity of their synaptic connections play a vital role in precisely regulating hippocampal network oscillations. Synaptic plasticity, such as long-term potentiation (LTP) and long-term depression (LTD), within these pathways may act as a fundamental mechanism for dynamically adapting network activity to environmental requirements or pathological circumstances. Exploring the plasticity of CCK → PC, BC → BC, BC → CCK and CCK → BC synapses in future research could offer more profound insights into how the hippocampal network responds to and compensates for disturbances. Additionally, while our model incorporates the same synaptic parameters and a uniform STDP rule for both thorny PCs and athorny PCs due to the current lack of subtype-specific plasticity data, emerging evidence suggests potential differences in their synaptic integration and plasticity properties. Future work should aim to investigate and integrate such heterogeneity, which may further refine our understanding of CA3 network adaptability. Such investigations could pave the way for a more comprehensive understanding of hippocampal network regulation and its broader implications for brain function.

In conclusion, this study provides important insights into the mechanisms by which inhibitory interneurons, specifically CCKs and BCs, regulate SWR generation and network oscillations in the CA3 region of the hippocampus. By highlighting the critical role of mutual inhibition between interneuron subtypes and its impact on network dynamics, our findings deepen the understanding of how inhibitory interactions shape hippocampal circuit function. These results not only advance our knowledge of the fundamental principles governing neural synchrony and SWR activity but also underscore the importance of interneuron diversity in a small-scale neural circuit model. This work lays a foundation for further theoretical and computational investigations into the role of inhibitory microcircuits in hippocampal information processing.

## Data Availability

The raw data supporting the conclusions of this article will be made available by the authors, without undue reservation.
